# The Risk of Aircraft-Acquired SARS-CoV-2 Transmission during Commercial Flights: A Systematic Review

**DOI:** 10.3390/ijerph21060654

**Published:** 2024-05-21

**Authors:** Diana Zhao, Stephanie Cheng, Fuchiang R. Tsui, Maya B. Mathur, Chih-Hung Jason Wang

**Affiliations:** 1 Center for Policy, Outcomes and Prevention, Department of Pediatrics, Stanford University School of Medicine, Stanford, CA 94305, USA; dyzhao@alumni.stanford.edu (D.Z.);; 2 Department of Anesthesiology and Critical Care Medicine, Children’s Hospital of Philadelphia, Philadelphia, PA 19146, USA; tsuif@chop.edu; 3 Quantitative Sciences Unit, Department of Pediatrics, Stanford University, Stanford, CA 94305, USA; mmathur@stanford.edu; 4 Center for Health Policy, Freeman-Spogli Institute for International Studies, Stanford University, Stanford, CA 94305, USA

**Keywords:** risk of aircraft-acquired SARS-CoV-2 transmission, COVID-19 risk on flights, coronavirus transmission on airplanes

## Abstract

The aircraft-acquired transmission of SARS-CoV-2 poses a public health risk. Following PRISMA guidelines, we conducted a systematic review and analysis of articles, published prior to vaccines being available, from 24 January 2020 to 20 April 2021 to identify factors important for transmission. Articles were included if they mentioned index cases and identifiable flight duration, and excluded if they discussed non-commercial aircraft, airflow or transmission models, cases without flight data, or that were unable to determine in-flight transmission. From the 15 articles selected for in-depth review, 50 total flights were analyzed by flight duration both as a categorical variable—short (<3 h), medium (3–6 h), or long flights (>6 h)—and as a continuous variable with case counts modeled by negative binomial regression. Compared to short flights without masking, medium and long flights without masking were associated with 4.66-fold increase (95% CI: [1.01, 21.52]; *p* < 0.0001) and 25.93-fold increase in incidence rates (95% CI: [4.1, 164]; *p* < 0.0001), respectively; long flights with enforced masking had no transmission reported. A 1 h increase in flight duration was associated with 1.53-fold (95% CI: [1.19, 1.66]; *p* < 0.001) increase in the incidence rate ratio (IRR) of cases. Masking should be considered for long flights.

## 1. Introduction

Air travel played an important role in the spread of the COVID-19 pandemic, as it facilitated the movement of potentially infected individuals across regions and continents [1]. To reduce the spread, governments and travelers responded by restricting, and in some cases stopping, travel. Yet reduced airline travel resulted in staggering financial losses: an estimated 370 billion USD loss in airline passenger operating revenues and a 200 billion USD loss in global tourism from January 2020 to January 2021 [2]. As of August 2020, governments had already injected over 160 billion USD into airlines in direct aid and wage subsidies to keep the aviation industry afloat [3]. While the aviation industry rebounded by the end of 2023 to reach 94% of the pre-pandemic traffic from 2019 [4], periods of high SARS-CoV-2 transmission have occurred annually and the prevention of SARS-CoV-2 transmission continues to be a public health priority [5].

The CDC has determined that the airborne transmission of SARS-CoV-2 tends to occur in environments with enclosed spaces and minimal distancing [6], both characteristic of aircraft cabins. Studies on airborne disease transmission demonstrate that in an enclosed environment where aerosol particles are well-mixed, the number of occupants, their time in the enclosed space, and mechanical ventilation are all important for the aerosol transmission of the SARS-CoV-2 virus in the COVID-19 pandemic [1,2,7]. Other studies find that the aircraft-acquired transmission of SARS-CoV-2 occurs despite airlines’ claims that HEPA filters and aircraft airflow are protective [8,9,10]. While the modes of transmission for SARS-CoV-2 are now well recognized, no synthesized information on flight risk has been established, particularly with regard to flight duration and strict masking. In this study, we aim to conduct a review of published reports of plausible and likely commercial aircraft-acquired SARS-CoV-2 transmission to identify factors that are important in aircraft-acquired transmission.

## 2. Materials and Methods

We conducted a qualitative and quantitative analysis of papers describing commercial flights during the COVID-19 pandemic, published between 24 January 2020 and 20 April 2021, before vaccines were available.

### 2.1. Data Sources and Searches

We first conducted a systematic review of the published literature using Scopus, the Web of Science, and LitCovid, a comprehensive central database updated daily with COVID-related literature from PubMed [11]. On 20 March 2021, we used the search term “airplane” for all years in LitCovid and “airplane” AND “COVID-19” OR “COVID” OR “coronavirus” in the Web of Science and Scopus. The Web of Science returned zero hits. To verify, our team did a rapid review of the Web of Science using a combination of the search terms “COVID-19”, “Covid,” and “coronavirus,” and confirmed there being no results. We revisited LitCovid and Scopus on 20 April 2021 with the added search terms OR “in-flight transmission” OR “aircraft transmission” OR “airplane transmission” for all years. Additionally, we employed a snowball search strategy for cited publications in the original hits, if relevant, with no restrictions on language or years.

### 2.2. Study Selection

We included articles if the flight had index cases and if they mentioned the flight duration or other identifying information that allowed us to determine the flight duration. We excluded articles discussing non-commercial aircraft or aircraft-related transport such as naval aircraft carriers, helicopters, or medical air ambulances; articles modeling SARS-CoV-2 transmission or airflow in aircrafts without flight data; articles discussing only medical details of COVID-19 patients without flight data; and articles where we were unable to determine whether in-flight transmission had occurred. We also excluded repatriation evacuation flights with medical staff on board because these flights do not translate to the commercial flight experience, patients are known to be infectious, and there are few fliers.

### 2.3. Data Extraction and Quality Assessment

Articles that met the inclusion and exclusion criteria were subjected to independent detailed review by at least two members of our research team (D.Z. or S.C.) and were discussed with a third member (C.J.W.) if any questions arose. For flights without an explicit flight duration, we conducted additional research using websites like Google to determine the approximate flight time. When possible, we used Seatguru to determine the number of passenger seats available. Coincidentally, all flights analyzed were direct flights.

We distinguished between “enforced masking” and “unenforced masking” on long-haul flights mentioning masking (8 flights) (see Table 1). We defined “enforced” masking as strict masking protocols implemented by airlines and flight attendants (6 of 8 flights). We defined “unenforced” masking as flights without a strict masking protocol but in which most passengers self-reported masking (2 of 8 flights). Unenforced masking was considered the same haul type as there being no masking at all in our analysis. Because long-haul flights included meals, and the only masking reported in our dataset was on long-haul flights, it can be assumed that both “enforced” and “unenforced” masking included mealtimes with masks off.

To minimize the risk of bias, we chose only flights where index cases were confirmed to ensure that the exposure (to an index case) temporally preceded the outcome (the transmission of SARS-CoV-2), meaning that there was a plausible temporal timeline for in-flight SARS-CoV-2 transmission [12]. We conducted a risk of bias assessment using a framework based on the ROBINS-I tool and calculated the E-value to assess the robustness to the potential uncontrolled confounding of the association between haul type and case incidence. The E-value represents the minimum strength of association on the risk ratio scale that unmeasured confounder(s) would need to jointly have with haul type and case incidence to fully explain away the results [13,14].

### 2.4. Data Synthesis and Analysis

In this study, we follow the guidelines of the Preferred Reporting Items for Systematic Reviews and Meta-Analyses (PRISMAs). To investigate the effect of flight duration on in-flight virus transmission, flights were grouped into haul types by duration, and analyzed by their ratio of infection. The ratio of infection is defined as the ratio of likely aircraft-acquired cases divided by the index cases on each flight, accounting for the effect of the number of index cases on virus transmission dynamics. Commercial flights categorize themselves into short-, medium-, and long-haul flights defined by less than 3 h, 3 to 6 h, and greater than 6 h of flight duration, respectively. We followed these commercial definitions and defined our flights into these three haul types for the ease of communication and because flights use these distinctions to determine what services to provide (e.g., snacks or meals). We added a fourth and separate flight haul type for enforced masking (n = 6).

To further explore the relationship between a continuous flight duration, the number of index cases, and the cases acquired in flight, we used the R statistical software (version 4.0.3) to perform negative binomial regression, a generalization of Poisson regression that accounts for overdispersion, to model the cases acquired in flight as a function of flight duration in hours. Index case count was incorporated into the negative binomial regression as an offset variable. Flights with enforced mask-wearing were omitted for this analysis, on the assumption that mask-wearing disrupted regular virus transmission. Several model diagnostics and sensitivity analyses were examined to investigate the fit of the negative binomial model.

Post hoc, we defined the studies with the “lowest risk” of bias as those that provided whole genome sequencing to trace transmission back to an in-flight index case. In whole genome sequencing, authors were able to identify the shared SARS-CoV-2 strain and confirm the strains to be 99% to 100% genetically identical to the strain of the index case. We considered studies to have a “moderately-low” risk of bias if they conducted standard contact tracing procedures, patient interviews, and the qt-PCR confirmation of SARS-CoV-2 infection. All studies we analyzed used at least one of these methods. Since all studies fell within the lowest to moderately low risk of bias, we did not conduct an extensive risk of bias analysis outside of calculating E-values.

## 3. Results

**Figure 1:**
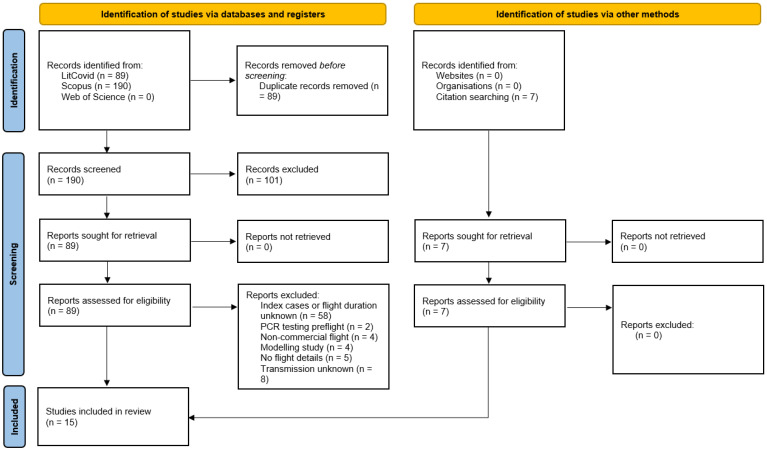
Study Selection.

A total of 96 unique articles were identified, with 89 being from the search terms and 7 being from our snowball strategy. A total of 58 articles did not meet our inclusion criteria. We excluded 21 articles (4 regarding non-commercial aircrafts, 4 for SARS-CoV-2 modeling, 5 for medical details, and 8 for undetermined transmission). Retrospectively, we excluded two more articles for PCR pretesting before the flight. There were 15 articles describing 50 flights that received a detailed review (see Figure 1). Individual flights were used as the unit of analysis.

**Figure 2:**
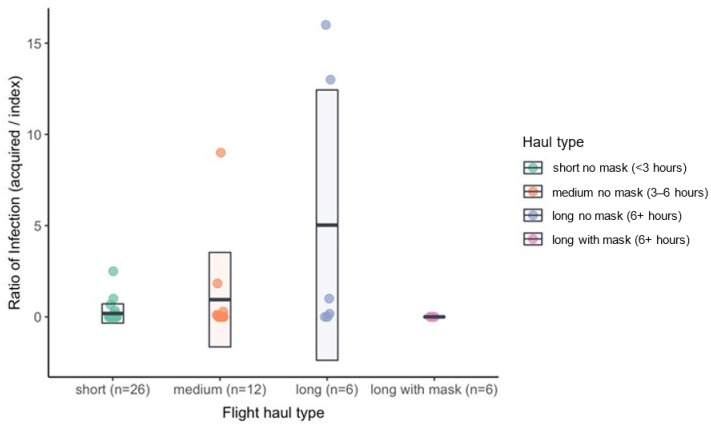
The aircraft transmission ratios by flight haul type (n = 50). Flights were categorized into haul types according to flight duration as follows: short (<3 h), medium (3–6 h), and long (6+ h). Cross bars show the median ratio of infection and boxes show the interquartile range. Though the majority of flights (n = 35; 70%) did not record any in-flight transmission, the remaining flights (n = 15, 30%) had a median ratio of infection of 0.67 (interquartile range [IQR]: 0.17, 2.17) if there was at least one acquired case. By haul type, the median ratio of infection among flights with at least one recorded transmission for short hauls without masking was 0.50 (IQR: 0.21, 0.92); for medium hauls without masking was 0.29 (IQR: 0.11, 1.83); and for long hauls without masking was 7.00 (IQR: 0.79, 13.75).

Of the 50 flights studied, the in-flight durations were as follows: 26 flights were short (ranging from 2 to 2.83 h long), 12 flights were medium (ranging from 3.5 to 5 h long), and 12 were long (ranging from 7.5 to 15 h long) (see Table 1). Most flights (n = 35; 70%) did not record any in-flight transmission (see Table 2 and Table 3). Among the remaining flights (n = 15, 30%) in which there was at least one acquired case, the median ratio of infection was 0.67 (interquartile range [IQR]: 0.17, 2.17) (see Figure 2).

By haul type, the numbers and percentages of flights with no transmission were, for short hauls without masking: 20 (77%); for medium hauls without masking: 7 (58%); for long hauls without masking: 2 (33%); and for long hauls with enforced masking: 6 (100%). By haul type, the median ratio of infection among flights with at least one recorded transmission were, for short hauls without masking: 0.50 (IQR: 0.21, 0.92); for medium hauls without masking: 0.29 (IQR: 0.11, 1.83); and for long hauls without masking: 7.00 (IQR: 0.79, 13.75) (see Figure 2).

**Figure 3:**
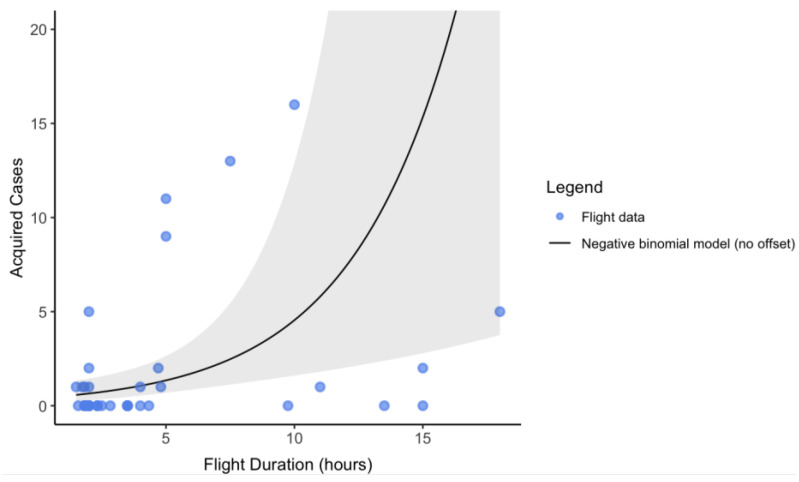
The cases acquired in flight, by flight duration. For visual simplicity, the negative binomial model is fitted without an offset.

In the negative binomial regression model, flight duration strongly predicted case incidence (see Figure 3). Compared to short flights without masking, medium flights without masking were associated with 4.66-fold increase in incidence rate (95% CI: [1.01, 21.52]; *p* < 0.0001); long flights without masking were associated with 25.93-fold increase in incidence rate (95% CI: [4.1, 164]; *p* < 0.0001); and long flights with enforced masking had no transmissions reported. As a continuous variable, a 1 h increase in flight duration was associated with 1.53-fold (95% CI: [1.19, 1.66]; *p* < 0.001) increase in the incidence rate of cases. Model fit diagnostics suggested the negative binomial model fit the data well.

As per the qualitative assessment, the risk of bias in our included studies was low. The estimated incidence rate ratio (IRR) appeared to be moderately robust to unmeasured confounding (E-value 2.43). With an observed IRR of 1.53 for continuous flight duration, only unmeasured confounder(s) jointly associated with both flight duration and SARS-CoV-2 infection by risk ratios of 2.43 each could potentially explain away the estimate, but confounder(s) with jointly weaker unmeasured confounding associations could not. With a lower confidence interval limit of 1.19, unmeasured confounder(s) jointly associated with both flight duration and SARS-CoV-2 infection by risk ratios of 1.66 each could potentially shift the confidence interval to include the null, but jointly weaker unmeasured confounding associations could not. In other words, a larger E-value indicates that it would take more unmeasured confounding to explain away the results of a study, and therefore that the study is more robust to potential unmeasured confounding [29].

## 4. Discussion

In this study, we found that the mean ratio of infection is associated with the duration of the flight when masking is unenforced. The ratio tends to be larger for longer flights compared to shorter flights. In addition, our negative binomial regression showed that flight duration strongly predicts case incidence. We also found that when masking is unenforced, each additional hour of flight duration is associated with 1.53-fold increase in the transmission incidence rate ratio. We speculate that short flights may be safer due to a shorter total duration of exposure to aerosol particles. Also, short flights often do not serve meals, so fewer aerosol particles and droplets are expelled. Interestingly, our findings also suggest that aircraft-acquired transmission is not inevitable if masking is strictly enforced. On long haul-type flights where enforced masking took place and meals were served, there were no reported aircraft-acquired cases during contact tracing and follow-up. Enforced masking may have encouraged passengers to eat as quickly as possible on these long flights. Furthermore, airline staff can actually enforce masking, similar to how staff are able to enforce safety checks such as correct table-up and seat up-and-back positions by walking down the aisles, checking each seat, and correcting behaviors during take-off and landing.

Strong evidence suggests that indoor transmissions drive the majority of COVID-19 spreader events, and, consistent with this fact, facemask directives have been more effective at controlling the spread of COVID-19 than lockdowns or social distancing [8]. Cumulative time spent indoors may also be important. There is ample evidence to support that COVID-19 spreads primarily through aerosol transmission [30,31]; aerosol particles containing infectious viruses can hang and accumulate in poorly ventilated indoor air [32]. This has public health implications: as asymptomatic and symptomatic individuals can release thousands of virus-laden aerosol particles when breathing and talking [31,33], reducing SARS-CoV-2 transmission requires reducing airborne transmission (such as via masking) whenever indoors [32].

Beyond our formal analysis, we observed as a point of interest that the proximity to the index case(s) was not the best predictor of aircraft-acquired transmission. For example, on a 2 h flight, one passenger seated five rows away acquired COVID [16]; on a 5 h flight, a passenger seated six rows away acquired COVID [22]; and on a 7.5 h flight and a 10 h flight, four passengers and one passenger who sat greater than 2 m (6 ft) away acquired COVID, respectively (see Table 3) [23,24].

Our findings have several limitations. First, our data came from a small sample of 50 flights describing likely aircraft-acquired SARS-CoV-2 infections; the data on air travel-related transmission were scant; most were observational studies without controls and there might be additional unpublished events. Second, we did not have the occupancy of each flight (i.e., how full the flights were). We estimated that all flights, unless otherwise noted, were likely to be one-third to two-thirds full, reasoning that airlines often decide to cancel flights if there are not enough passengers, and that most airlines blocked middle seats during this period of the pandemic for social distancing. Third, we cannot exclude other risks in air travel beyond in-flight risks, e.g., queuing for security or customs or boarding the plane, as well as the waiting time on the runway or transfers to terminals in public buses. Fourth, although it is striking that the six masked flights had no transmissions at all, it is unclear whether mask-wearing was the only or direct cause of this, since it is possible that flights with masking protocols also implemented other safety measures (e.g., minimizing boarding times). Fifth, our results cannot separate whether there is something distinctive about long flights themselves or the subset of the population taking long flights that contributes to SARS-CoV-2 transmission. Sixth, the data analyzed were collected during the period of the original Wuhan variant, not the more contagious Delta or Omicron variants, and prior to the existence of vaccinations, which means our results could have underestimated the effect of long flight durations on transmission.

## 5. Conclusions

Our paper is one of the first to statistically show that a longer flight duration is associated with a greater risk of SARS-CoV-2 transmission from empirical commercial flight data. Stakeholders can and should re-evaluate their safety policies for fliers in the context of existing policies such as those regarding full-density flights (middle seats ceased to be blocked on 1 May 2021) [34], providing proof of negative COVID tests for international travelers (required by the United States on 17 December 2021) [35], meal practices, and masking and sanitation policies [3]. Flight policies regarding masking based on travel duration may become important for air travel safety in future epidemics or pandemics, particularly before effective vaccines or medications are made available.

## Figures and Tables

**Table 1:** The flight haul type breakdown.

Flight Haul Type	Number of Flights	Masking Mentioned on Flights	Enforced Masking
Short (<3 h)	26	0	0
Medium (3–6 h)	12	0	0
Long (>6 h)	12	8	6
Total flights	50	8	6

**Table 2:** The flights ordered by duration.

Flight Number	Approximate Flight Date	Flight Duration (h)	Index Cases (per Flight)	Aircraft-Acquired Cases (per Flight)	Masking
1	24 February 2020	1.5	1	1	Unenforced
2	31 January 2020 to 3 December 2020	1.58	1	0	Unenforced
3		1.75	7	1	Unenforced
4		1.83	2	0	Unenforced
5		1.83	2	0	Unenforced
6		1.83	2	0	Unenforced
7		1.83	2	0	Unenforced
8		1.83	9	0	Unenforced
9		1.83	3	1	Unenforced
10		2	3	0	Unenforced
11		2	3	2	Unenforced
12		2	6	1	Unenforced
13		2	3	0	Unenforced
14		2	5	0	Unenforced
15		2	1	0	Unenforced
16		2	1	0	Unenforced
17		2	2	0	Unenforced
18		4	2	0	Unenforced
19		4.33	1	0	Unenforced
20	27 February 2020	2	2	5	Unenforced
21	1 March 2020	2	1	0	Unenforced
22	3 March 2020	2	1	0	Unenforced
23	6 March 2020	2	1	0	Unenforced
24	22 February 2020	2.33	1	0	Unenforced
25	23 February 2020	2.33	1	0	Unenforced
26	26 February 2020	2.33	1	0	Unenforced
27	26 February 2020	2.42	2	0	Unenforced
28	28 February 2020	2.83	1	0	Unenforced
29	3 March 2020	3.5	1	0	Unenforced
30	4 March 2020	3.5	1	0	Unenforced
31	7 March 2020	3.5	1	0	Unenforced
32	8 March 2020	3.5	1	0	Unenforced
33	8 March 2020	3.5	2	0	Unenforced
34	1 March 2020	9.75	1	0	Unenforced
35	24 January 2020	4	9	1	Unenforced
36	9 March 2020	4.7	7	2	Unenforced
37	24 January 2020	4.8	12	1	Reported
38	24 January 2020	5	1	9	Unenforced
39	19 March 2020	5	6	11	Unenforced
40	Summer-2020	7.5	1 (likely)	13	Unenforced
41	2 March 2020	10	1	16	Unenforced
42	31 March to 1 April 2020	11	6	1	Unenforced
43	9 to 10 March 2020	15	2	2	Unenforced
44	22 January 2020	15	1	0	Reported
45	16 June 2020	8	6	0	Mandatory, Enforced
46	21 June 2020	8	29	0	Mandatory, Enforced
47	23 June 2020	8	16	0	Mandatory, Enforced
48	3 July 2020	8	9	0	Mandatory, Enforced
49	4 July 2020	8	7	0	Mandatory, Enforced
50	20 February 2020	13.5	2	0	Mandatory, Enforced

Footnote: Lines separate articles and correspond to our comments in Table 3.

**Table 3:** Observations by flight number.

Flight Number	Study Design	Observations
1	Case report (retrospective)	From Bangui to Yaounde [15]
2 to 19	Retrospective cohort study	From Innsbruck, Berlin, Milan, Turin, Verona, Tenerife, Basel, Geneva, and Istanbul to England. Of the aircraft-acquired COVID-19 cases, 4 passengers sat within 2 seats of the index case, and 1 sat 5 rows away of the index case. There were a total of 55 index cases among 18 flights, which led to 5 aircraft-acquired cases. Flights averaged 1–7 index cases [16].
20 to 34	Retrospective	From Northern Italy, Israel, and the UK to Greece, as well as departures to and from Greece. On a flight from Israel to Athens (27 February 2020), 5 aircraft-acquired cases occurred in close contact with an index case (within 2 seats) and less than 2 m away for greater than 15 min. 1 case was a flight attendant, while 4 were passengers [17].
35	Retrospective	From Malaysia to Hangzhou. 3 index cases were symptomatic, and 6 index cases were asymptomatic [18].
36	Retrospective	From Tel Aviv, Israel to Frankfurt, Germany. The aircraft-acquired cases were seated within 2 rows of the index case [19].
37	Retrospective	From Singapore to Hangzhou. The aircraft-acquired case was seated next to 4 infectious passengers. Meals were served. A total of 16 passengers tested positive after the flight, but not everyone who tested positive was interviewed [20].
38	Retrospective	From Singapore to Hangzhou. There were 2 additional likely aircraft-acquired cases who the authors were unable to interview [21].
39	Retrospective	From Sydney to Perth, Australia. For the aircraft-acquired cases, 8 passengers sat within 2 rows of the index cases, 2 passengers sat 3 rows away, and 1 was 6 rows away. Of the 11 aircraft-acquired cases, the authors categorized 8 as confirmed and 3 as probable [22].
40	Retrospective	From 3 different continents (unspecified) into Ireland. Of the aircraft-acquired cases, 9 sat in a close contact range to the index case while 4 fell outside a close contact range (greater than 2 m). 9 out of 13 (69%) of the aircraft-acquired cases self-reported mask-wearing [23].
41	Retrospective	From London to Hanoi. Of the aircraft-acquired cases, 13 were in business class with the index case, while 2 passengers and 1 flight attendant were in economy class. Among those in business class, 11 were within 2 m of index case (2 seats), while 1 was greater than 2 m away. Aircraft-acquired cases in economy class fell out of a close contact range to the index case [24].
42	Prospective	From Milan, Italy to South Korea. An aircraft-acquired case was seated 3 rows away from an index case, and shared a bathroom with the index case. Index cases were asymptomatic [25].
43	Retrospective	From Boston, Massachusetts to Hong Kong. 2 aircraft-acquired cases were flight attendants who served index cases [26].
44	Letter, retrospective	From Wuhan, China to Guangzhou, China to Toronto, Canada. No transmission. The index case was mild [27].
45–49	Review (prospective, retrospective)	From Dubai to Hong Kong. No transmission. All infected passengers originated from Pakistan. Emirates flights were known to be rigorous with their mask enforcement [10].
50	Prospective	From Japan to Israel. No transmission. Passengers took off masks when eating meals, for ~15 min each time. There were 2 meals served on the flight. There were only 11 passengers on the flight [28].

## Data Availability

The data presented in this study are available on request from the corresponding author.
